# The Role of Neutrophils and Neutrophil Extracellular Traps in Acute Pancreatitis

**DOI:** 10.3389/fcell.2020.565758

**Published:** 2021-01-21

**Authors:** Jianhua Wan, Yuping Ren, Xiaoyu Yang, Xueyang Li, Liang Xia, Nonghua Lu

**Affiliations:** ^1^Department of Gastroenterology, The First Affiliated Hospital of Nanchang University, Nanchang, China; ^2^Department of Rheumatology, The First Affiliated Hospital of Nanchang University, Nanchang, China

**Keywords:** neutrophil, pancreatitis, neutrophil granules, NETs, inflammation

## Abstract

Leukocyte invasion (neutrophils and monocytes/macrophages) is closely related to the severity of acute pancreatitis (AP) and plays an important role in the systemic inflammatory response and other organ injuries secondary to AP. Increased and sustained activation of neutrophils are major determinants of pancreatic injury and inflammation. After the onset of AP, the arrival of the first wave of neutrophils occurs due to a variety of triggers and is critical for the exacerbation of inflammation. In this review, we summarize the functional characteristics of neutrophils, elastase, and heparin-binding proteins in granules, the mechanisms of neutrophil recruitment and the role of neutrophil extracellular traps (NETs) in AP.

## Introduction

Neutrophils are polynuclear white blood cells and one of the main responders to acute inflammation. White blood cells are usually the first cell type recruited to the site of inflammation and fight pathogens through a variety of mechanisms. In humans, neutrophils account for 50–70% of all circulating white blood cells, while in mice, neutrophils account for 10–25% of circulating white blood cells (Doeing et al., 2003; Mestas and Hughes, 2004). In circulation, the nuclei of mature neutrophils are segmented and approximately 7–10 μm in diameter, and the cytoplasm is rich in granules and secretory vesicles (Borregaard, 2010). Neutrophils are the first responders in host defense against a variety of pathogens, including bacteria, fungi, and protozoa. The migration of neutrophils to the site of inflammation is essential for clearing the infection, and a significant reduction in the number of neutrophils in the blood can lead to severe immune deficiency. In addition to clearing pathogens in acute infections, neutrophils play important and adverse roles in other inflammation-related diseases.

Acute pancreatitis (AP) is pancreatic inflammation caused by abnormal activation of trypsinogen, which can lead to systemic inflammatory response syndrome (SIRS) and organ failure (Lankisch et al., 2015). In particular, acute lung injury (ALI) can be observed at an early stage in patients with severe acute pancreatitis (SAP) (Zhou et al., 2010). Although there has been considerable progress in recent years in the treatment of AP, especially SAP, the mortality rate is still high, and the specific pathogenesis is still unclear. Typical features of pancreatic injury and a large number of inflammatory cells, including neutrophils and macrophages, have been observed in pancreatic tissue specimens from patients with AP and animal models. Neutrophils are primarily thought to be the first immune cells to be recruited to inflammatory tissue in the event of acute inflammation. Neutrophils are important in systemic injury and death associated with SAP that is induced by choline-deficient ethionine-supplemented (CDE) diet L-arginine or deoxycholic acid injection into the pancreatic duct. Neutrophil depletion can significantly reduce pancreatic injury and damage to organ systems and significantly improve the SAP survival rate (Inoue et al., 1995; Kyriakides et al., 2001; Chen et al., 2015). In addition, neutrophils mediate further activation of trypsinogen byproducts of NADPH oxidase, exacerbate pancreatic injury and even cause lung injury (Gukovskaya et al., 2002; Abdulla et al., 2011). Thus, the purpose of this review is to further discuss the function of neutrophils and the pathogenesis of AP.

## Features and Functions of Neutrophils

Neutrophils are derived from myeloid progenitor cells in the bone marrow and are regulated by many transcription factors, proteins, and receptors. The main neutrophil regulator is granulocyte colony stimulating factor (G-CSF) (Lieschke et al., 1994). Neutrophils have been regarded as having a short lifespan of 6–8 h in humans and mice, but they have also been reported to live up to 5.4 days (Pillay et al., 2010; Tak et al., 2013). However, during the inflammatory response, neutrophils are stimulated and activated, and their lifespan is significantly increased several-fold, thus ensuring the persistence of neutrophils at the site of infection (Colotta et al., 1992; Summers et al., 2010). Neutrophils have many biological functions, including chemotaxis, antimicrobial functions, phagocytosis, degranulation, and neutrophil extracellular trap (NET) production. *In vivo*, neutrophils are the first line of defense against bacterial and fungal infections. These cells kill invading pathogens with a large number of antibacterial agents, including reactive oxygen species (ROS) and hydrolases. Neutrophils release NETs and antibacterial granules through NETosis to kill and prevent the spread of pathogens (Brinkmann et al., 2004). It is well-known that during acute inflammation, neutrophils are rapidly recruited to tissues affected by inflammation in a multistep recruitment cascade (Maas et al., 2018). Although neutrophil recruitment is traditionally thought to be unidirectional (from blood to tissue), some studies have also shown that neutrophils can migrate from tissue to blood and even other tissues, causing secondary tissue damage (Woodfin et al., 2011; Wang et al., 2017).

## Neutrophil Recruitment in Acute Pancreatitis

### Neutrophil Recruitment to Pancreas During AP

During an inflammatory response, the recruitment cascade of leukocytes occurs and involves tethering, rolling, adhesion, crawling, and eventually migration. Initial neutrophil recruitment cascades on the surface of endothelial cells result from stimulation by inflammatory mediators (including cytokines, leukotrienes, and histamines) that are released by tissue-resident leukocytes as a result of encounters with pathogens (Ley et al., 2007; Phillipson and Kubes, 2011; Sadik et al., 2011). Large doses of cerulein induce a significant increase in the expression of CXCL2 (also known as macrophage inflammatory protein-2), which is an effective neutrophil attractant (Pastor et al., 2003). Decreased levels of CXCL2 in the pancreas in AP reduce pancreatic neutrophil infiltration and tissue damage and reduce neutrophil invasion in lung tissue (Merza et al., 2013). Similarly, platelet-derived CXCL4 regulates neutrophil recruitment via secretion of CXCL2, and inhibition of CXCL4 markedly decreases pancreatic injury and plasma levels of CXCL2 in animals with AP (Wetterholm et al., 2016). When chemokines bind to polymorphonuclear leukocytes (PMNs), the intracellular store of MAC1 is also relocated to the cell surface (Jones et al., 1988). Deletion of CD18, a subunit of the MAC-1 complex, prevents transmigration of neutrophils into the pancreas during pancreatitis, greatly reducing disease severity and decreasing intrapancreatic protease activation (Sendler et al., 2013). The binding of lymphocyte function antigen-1 (LFA-1) to immunoglobulin-like cell adhesion molecule 1 (ICAM1) is essential for neutrophils to adhere firmly to endothelial cells (Phillipson et al., 2006). LFA-1 plays a key role in the regulation of pancreatic neutrophil recruitment, CXCL2 formation and tissue damage in taurocholate-induced AP (Awla et al., 2011). The interaction between PMNs and the endothelium is mediated by specific adhesion molecules in the selectin and integrin families, and adhesion also leads to enhanced activation of the neutrophil signaling pathway. Inhibition of P-selectin alleviated tissue damage by reducing neutrophil-endothelium interactions and improving pancreatic microcirculation in experimental AP (Hackert et al., 2009; Hartman et al., 2012). Neutrophils began to show adaptive changes in morphology, with actin cytoskeleton rearrangement (Leick et al., 2014). The neutrophils began to extend foot processes across the entire endothelial cell surface and then to move.

### Neutrophil Recruitment to Lung During AP

Neutrophils are the first cells of the immune system to collect at damaged or inflammatory sites and are believed to play a key role in the development of AP-associated ALI (Willemer et al., 1991). Lung injury is caused by local lung endothelial cell injury secondary to the production of oxygen free radicals by neutrophils (Guice et al., 1989). During AP, a large number of neutrophils are rapidly recruited from the peripheral circulation to the site of pancreatic inflammation. Toll-like receptor 4 (TLR4) mediates the immune response and plays a proinflammatory role in the progression of acute pancreatitis by promoting neutrophil recruitment (Sharif et al., 2009). The neutrophils that accumulate in the pancreas migrate in a retrograde manner to the circulatory system, causing systemic inflammation and damage to other organs. Circulating neutrophils isolated from patients during AP cause endothelial damage (Paulino et al., 2007). These cells are tissue-characteristic neutrophils, have distinct phenotypes and are known as reverse-migrating neutrophils. Reverse-migrating neutrophils have a more robust ability to produce ROS than other types of neutrophils, thereby exacerbating local tissue damage. Knockdown of junctional adhesion molecule-C (JAM-C) exacerbates lung injury and systemic inflammation in experimental pancreatitis. An increase in neutrophil reverse transendothelial migration (rTEM) was observed in JAM-C-deficient mice, and JAM-C played a key role in promoting rTEM (Wu et al., 2016). Neutrophils that reach the basement membrane pause before crossing the basement membrane. Matrix metalloproteinase 9 (MMP-9) is necessary for neutrophil migration by degrading some basement membrane components, including type IV collagens, fibronectins, and gelatin. In a model of severe necrotizing pancreatitis, MMP-9 is released from neutrophils stimulated by pancreatic enzymes and proinflammatory mediators. Inhibition of MMP-9 by batimastat (BB-94) reduces neutrophil transmigration and alveolar capillary permeability in pancreatitis-associated lung injury (Keck et al., 2002).

## The Proteins in Neutrophil Granules in Acute Pancreatitis

Neutrophils also release granules that have antimicrobial properties in the progression of many inflammatory disorders via a variety of mechanisms. Granules include primary neutrophil granules, which are composed of elastase, cathepsin G, myeloperoxidase (MPO), and other proteins, and secondary and tertiary granule particles, which are composed of proteins such as lactoferrin and gelatinase (Papayannopoulos et al., 2010; Metzler et al., 2014).

### Neutrophil Elastase

Analysis of serum samples of AP patients in the clinic showed that the activity of neutrophil elastase (NE) was associated with the predicted severity of AP and AP-associated respiratory failure (Novovic et al., 2013). During the early pathological process of pancreatitis, the dissolution of cell-cell contacts causes the formation of pancreatic edema and cell damage. While E-cadherin is the key protein mediating cell contact, neutrophil elastin cleaves E-cadherin to cause the dissociation of cell contacts, which then permits increased transmigration of leukocytes into the pancreas (John et al., 2019). Inhibition of NE *in vivo* significantly reduced the translocation of leukocytes and decreased disease severity (Mayerle et al., 2005; Jo et al., 2008). Neutrophils with increased NE activity exacerbate pulmonary edema and damage in ALI (Kinoshita et al., 2000). NE is not only involved in pancreatic tissue injury but also aggravates the systemic inflammatory response, thus causing the critical process of AP. Therefore, effective targeted inhibition of NE may be a promising strategy for the treatment of AP, thereby reducing the incidence of severe disease.

### Cathepsin G

Cathepsin G (CTSG) is a serine protease of the chymotrypsin C family that is stored in primary granules of neutrophils. Its major biological functions include the degradation of extracellular matrix and plasma proteins. The broad serine proteinase inhibitor (serpin) Lex032, which can inhibit both CTSG and NE, reduces the capillary no-reflow phenomenon and pancreatic tissue damage in ischemia/reperfusion-induced pancreatitis (von Dobschuetz et al., 1999). However, Aghdassi et al. found that CTSG knockout affects neutrophil invasion in early AP but does not affect the severity of the disease because CTSG had no effect on intraacinar cell trypsinogen activation (Aghdassi et al., 2019). Compared with targeted inhibition of NE activity, inhibition of CTSG does not seem to have a promising effect in the experimental AP model.

### Heparin-Binding Protein

Human neutrophils secrete cationic antimicrobial protein 37, which efficiently binds heparin and is also named heparin-binding protein (HBP). HBP plays an important role in regulating inflammation and promoting vascular leakage (Gautam et al., 2001; Soehnlein et al., 2008). Through network bioinformatics analysis, Nune et al. found that among normal healthy people and patients with pancreatic diseases, the extracellular HBP concentration coefficient was highest in AP patients (Nunes et al., 2013). Glycosaminoglycan, as an inhibitor of HBP, can reduce the occurrence of cerulein-induced AP by reducing the inflammatory response and even improve the survival rate of the sepsis mouse model (Juhas et al., 2018). In addition, many studies have shown that serum HBP levels are positively correlated with the severity of many critical inflammatory diseases and can be used as a promising serum marker to predict disease severity (Linder et al., 2009; Lin et al., 2013; Tyden et al., 2017). Based on the characteristics of HBP, it was hypothesized that HBP is involved in the process of severe disease and the functional injury of organs other than the pancreas in SAP.

### Neutrophil Extracellular Traps in Acute Pancreatitis

Neutrophils have a key role in combating bacterial infections and have evolved additional mechanisms that increase their effectiveness in eliminating pathogens. NETs are the second bactericidal mechanism of neutrophils (Brinkmann et al., 2004). NETs are composed of decondensed DNA complexed with histones and granulosa proteins and do not contain any other cytoskeletal proteins. Nucleic acid substances are the backbone of NETs and form a skeletal structure that holds various protein particles together. NETs are produced by a variety of inducing factors, such as ROS, IL-8, LPS, complement factor 5 a, and β-glucan (Delgado-Rizo et al., 2017). ROS production is the key to PMN outbreaks and NET release (Stoiber et al., 2015). NETs trap and kill microbes, amplify immune responses, and induce coagulation (Sollberger et al., 2018). There are three important enzymes in NET production: peptidyl arginine deiminase 4 (PAD4), NE and MPO. Calcium ions can activate PAD4, which catalyzes the transformation of histone arginine into citrulline, reducing the strong positive charge of histones and weakening the binding of histones to DNA to promote chromatin depolymerization, which is the basis for NET formation *in vivo* (Demaurex et al., 1994; Wang and Wang, 2013; Sorensen and Borregaard, 2016). After chromatin depolymerization, the nuclear membrane ruptures, and chromatin is released into the cytoplasm, which is then released into the extracellular space through modification by granular protein to form NETs (Metzler et al., 2014). Even when stimulated by pathogenic activators such as bacteria, PAD4 knockout mice were unable to form NETs (Li et al., 2010). Therefore, the PAD4-induced deimination of histones is a prerequisite for the formation of NETs *in vivo*. Chlor-amidine irreversibly inhibited PAD4 through covalent modification at the active site of the enzymes and prevented NET-mediated vascular damage, endothelial dysfunction, and kidney injury in a mouse model of lupus (Knight et al., 2015; Witalison et al., 2015). NETs are a physical barrier that limits the spread of pathogens and inflammatory transmitters, preventing damage to normal tissues. NETs seem to play a protective role in the necrotic areas of acute necrotizing pancreatitis and form a provisionary tissue barrier separating necrotic areas from the remaining viable tissue (Bilyy et al., 2016). NETs can kill pathogens and are related to nucleic acid substances, NE, antimicrobial peptides and other components. The DNA skeleton makes NETs highly bactericidal, and the phosphodiester backbone of DNA is critical for this bactericidal activity (Halverson et al., 2015). Although NETs play a role in killing microbes, they have some negative effects, such as stimulating autoimmunity and thrombosis (Sorensen and Borregaard, 2016).

NETs play an important role in the pathophysiological mechanism of AP by promoting pancreatic tissue damage in a variety of ways. In a mouse model of acute necrotizing pancreatitis (ANP) induced by sodium taurocholate, a large amount of NETs and histones were deposited in the pancreatic tissue (Merza et al., 2015). NETs induce the activation of trypsinogen through STAT3, MMP-9, and other pathways, thereby amplifying the degree of pancreatic injury (Awla et al., 2012; Zhang et al., 2013; Korhonen et al., 2015; Merza et al., 2015). The clearance of neutrophils significantly reduces NET deposition in pancreatic tissue and cell-free DNA levels in plasma. Deoxyribonuclease I (DNase I) can depolymerize the DNA skeleton in NETs and thus destroy the structure of NETs (Meng et al., 2012). After DNase I administration, the expression level of histones in the pancreatic tissue of SAP mice decreased, the degree of pancreatic edema significantly decreased, and the level of blood amylase was also reduced (Merza et al., 2015). Chloroquine, as an autophagy inhibitor, improves the prognosis of mice with AP by reducing NET formation and the levels of serum cell-free DNA and citrullinated histone H3 (Murthy et al., 2019). In addition, one study revealed another mechanism by which the accumulation of NETs in the pancreatic duct leads to catheter obstruction, which drives pancreatic inflammation, while PAD4 deficiency prevents disease progression by reducing the formation of NETs in the duct (Leppkes et al., 2016). Therefore, targeted inhibition of NETs is expected to be a clinical strategy for the treatment of AP.

Local and systemic early pathological events of AP are associated with vascular disorders, including endothelial activation and injury, increased vascular permeability, and coagulation activation (Dumnicka et al., 2017). NETs promote thrombosis and have been shown to trap large concentrations of platelets, activated thrombin, and fibrin clots in a mouse model of sepsis. Similarly, the removal of NETs by DNase I can significantly decrease thrombin activity, reduce platelet aggregation, and improve microvascular permeability (McDonald et al., 2017). NETs provide a physical scaffold for thrombosis (Fuchs et al., 2010). Furthermore, platelet inositol hexaphosphatase 1 (IP6K1) is a key NET formation gene. Thrombin stimulation of IP6K1-deficient platelets in the presence of neutrophils results in no NET formation. NET formation induced by taurocholate reduced pancreatic inflammation and tissue damage in IP6K1-deficient mice (Madhi et al., 2019). Therefore, NETs play a role in mediating inflammation and thrombosis, bridging the transition between hypercoagulability and severe inflammation in the early pathophysiology of pancreatitis. A recent study found that dabigatran, an anticoagulant and antitrypsin drug, has a promising synergistic effect in mice with severe pancreatitis (Gui et al., 2020).

The systemic inflammatory response is the main manifestation of SAP, and respiratory failure is the most common complication of SAP. In the serum of SAP patients, the levels of cell-free DNA and DNA-histone complexes were significantly elevated. Circulating NETs play an important role in the recruitment of neutrophils in the lung, resulting in lung injury (Merza et al., 2015). NET structures were detected in the blood of patients with transfusion-related acute lung injury (TRALI), and NETs were also observed in the TRALI mouse model; NET formation was alleviated by DNA enzyme I treatment (Caudrillier et al., 2012). This finding indicates that NETs cause lung epithelial cell injury, and the targeted formation of NETs may be a new strategy for the treatment of lung injury in SAP.

### Perspectives of Anti-neutrophil Treatments in AP

In the early stage of acute pancreatitis, sterile inflammation is present, which induces systemic inflammatory response syndrome (SIRS), and the recruitment and activation of neutrophils play a major role (Sendler et al., 2020). The use of glucocorticoids and immunosuppressive agents in acute pancreatitis is still controversial, mainly because of the subsequent compensatory anti-inflammatory response syndrome (CARS) (Dong et al., 2015; Sendler et al., 2020). In the early stage of AP, neutrophils play an important proinflammatory role as the first recruit of acute inflammatory cells. Depletion of neutrophils by anti-Gr-1 antibody relieved plasma amylase and MPO activities, inflammation and tissue damage in the pancreas and lung in an L-arginine, choline-deficient ethionine (CDE) diet, or taurocholate-induced AP mouse model (Bhatia et al., 1998; Abdulla et al., 2011; Chen et al., 2015). Although one study showed that neutrophil depletion by injection of anti-Gr1 resulted in blockade of intracapillary leucocyte accumulation and led to the development of severe acute pancreatitis with fulminant intrapancreatic hemorrhage in taurocholate or enterokinase pancreatitis (Ryschich et al., 2009), intracapillary leucocyte accumulation was not observed in other models of acute pancreatitis, and decreasing leucocyte extravasation exhibited a protective effect. Platelet activating factor, an endogenous lipid mediator of inflammation, mediates the activation and infiltration of neutrophils into the tissue, and administration of its antagonist abolished apoptosis in experimental models of pancreatitis (Sandoval et al., 1996). In the early stages of AP, activated neutrophils release proteins in neutrophil granules, and NETs cause pancreatic inflammation and injury and even a systemic inflammatory response. Pharmacological inhibition of neutrophil biological behavior or the production of neutrophil products has become a treatment strategy for acute pancreatitis.

## Conclusions and Future Directions

**Figure 1:**
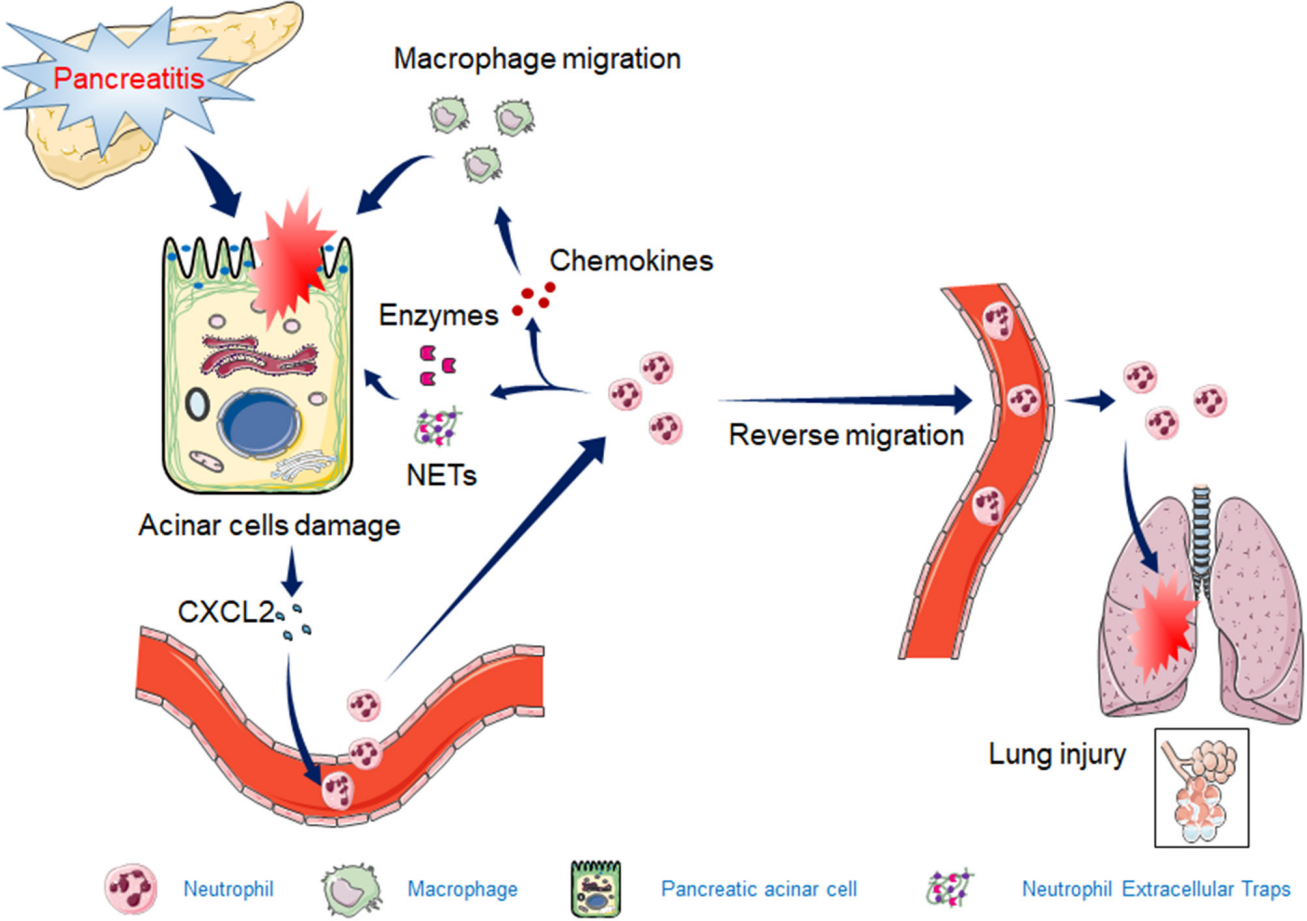
The mechanism by which neutrophils exacerbate acute pancreatitis (AP). The release of CXCL2 in early AP tissues induces the recruitment of neutrophils. Activated neutrophils induce the release of granulocytes, NET formation, and macrophage recruitment by releasing inflammatory chemokines, leading to increased pancreatic injury. Some of the activated neutrophils reverse migrate to the circulatory system, causing acute lung injury.

Total neutrophil deficiency or a significant reduction in the number of neutrophils can significantly reduce AP injury and inflammation. However, the reduction in neutrophils in mice is often associated with immunodeficiency. During the process of AP, neutrophils in pancreatic tissue appear to be overactivated or inappropriately recruited, resulting in pancreatic injury and even lung tissue damage. Prophylactic rectal non-selective non-steroidal anti-inflammatory drugs (NSAIDs) reduce the incidence or severity of post-ERCP (endoscopic retrograde cholangiopancreatography) pancreatitis, which reduces neutrophil infiltration into the pancreas (Geng et al., 2020). In summary, this review showed that neutrophil recruitment, granulocyte release, and NET formation are all involved in the exacerbation of AP (Figure 1). With the discovery of a new mechanism of neutrophils in AP, more precise or targeted therapy can be used to limit neutrophil-mediated destruction, minimize pancreatic injury and systemic inflammation, and minimize damage to patient immunity.

## Author Contributions

JW and YR conceived the study, participated in the design, and drafted the manuscript. XY, XL, LX, and NL edited and checked the manuscript. All of the authors have read and approved the final manuscript.

## Conflict of Interest

The authors declare that the research was conducted in the absence of any commercial or financial relationships that could be construed as a potential conflict of interest.

## Abbreviations

AP: acute pancreatitis
NETs: neutrophil extracellular traps
SIRS: systemic inflammatory response syndrome
ALI: acute lung injury
SAP: severe acute pancreatitis
CDE: choline-deficient ethionine-supplemented
G-CSF: granulocyte colony stimulating factor
ROS: reactive oxygen species
PMN: polymorphonuclear leukocyte
LFA-1: lymphocyte function antigen-1
ICAM1: immunoglobulin-like cell adhesion molecule 1
JAM-C: junctional adhesion molecule-C
rTEM: reverse transendothelial migration
MMP-9: matrix metalloproteinase 9
MPO: myeloperoxidase
NE: neutrophil elastase
CTSG: Cathepsin G
HBP: heparin-binding protein
PAD4: peptidyl arginine deiminase 4
ANP: acute necrotizing pancreatitis
DNase I: Deoxyribonuclease I
IP6K1: platelet inositol hexaphosphatase 1
TRALI: transfusion-related acute lung injury
CARS: compensatory anti-inflammatory response syndrome
CDE: choline-deficient ethionine
NSAIDs: non-steroidal anti-inflammatory drugs.
